# Natural Aromatic Compounds as Scaffolds to Develop Selective G-Quadruplex Ligands: From Previously Reported Berberine Derivatives to New Palmatine Analogues

**DOI:** 10.3390/molecules23061423

**Published:** 2018-06-12

**Authors:** Marco Franceschin, Lorenzo Cianni, Massimo Pitorri, Emanuela Micheli, Stefano Cacchione, Claudio Frezza, Mauro Serafini, Ming-Hao Hu, Huafi Su, Zhishu Huang, Lianquan Gu, Armandodoriano Bianco

**Affiliations:** 1 Dipartimento di Chimica, Università di Roma “La Sapienza”, Piazzale Aldo Moro 5, 00185 Rome, Italy; lorenzo.cianni@usp.br (L.C.); pitorrimassimo@hotmail.it (M.P.); armandodoriano.bianco@uniroma1.it (A.B.); 2 Dipartimento di Biologia e Biotecnologie, Università di Roma “La Sapienza”, Piazzale Aldo Moro 5, 00185 Rome, Italy; emanuela.micheli@uniroma1.it (E.M.); stefano.cacchione@uniroma1.it (S.C.); 3 Dipartimento di Biologia Ambientale, Università di Roma “La Sapienza”, Piazzale Aldo Moro 5, 00185 Rome, Italy; mauro.serafini@uniroma1.it; 4 School of Pharmaceutical Sciences, Shenzhen University Health Science Center, Shenzhen 518060, China; humhao1229@szu.edu.cn; 5 School of Pharmaceutical Sciences, Sun Yat-sen University, Guangzhou 510275, China; huafisu@sina.com (H.S.); ceshzs@mail.sysu.edu.cn (Z.H.); cesglq@mail.sysu.edu.cn (L.G.)

**Keywords:** G-quadruplex DNA, interactions, berberine and palmatine derivatives, NMR, FRET and MST assays

## Abstract

In this paper, the selective interactions of synthetic derivatives of two natural compounds, berberine and palmatine, with DNA G-quadruplex structures were reported. In particular, the previous works on this subject concerning berberine were further presented and discussed, whereas the results concerning palmatine are presented here for the first time. In detail, these palmatine derivatives were developed by inserting seven different small peptide basic chains, giving several new compounds that have never been reported before. The preliminary studies of the interactions of these compounds with various G-quadruplex-forming sequences were carried out by means of various structural and biochemical techniques, which showed that the presence of suitable side chains is very useful for improving the interaction of the ligands with G-quadruplex structures. Thus, these new palmatine derivatives might act as potential anticancer drugs.

## 1. Introduction

A G-quadruplex is a helical tertiary structure of DNA and RNA that is formed by sequences that are particularly rich in guanine through the stacking of at least two guanine tetrad subunits. At their own time, each of these subunits is generated by the association of four guanines in a square planar geometry that is internally stabilized by Hoogsteen hydrogen bonds, and externally stabilized by a cation (prevalently sodium and potassium) that is present in the physiological environment [1,2]. 

G-quadruplex structures (GqS) have been widely shown to exist in the genome and in the transcriptome including human telomeres [3], oncogenic promoters [4,5,6], and the 5′-untranslated region (5′-UTR) of mRNAs [7].

Nevertheless, it has been demonstrated that GqS can inhibit the activity of telomerase, which is an enzyme that adds telomeric repeats to the ends of chromosomes, and helps cancer proliferation [8]. GqS can also suppress the expression of specific oncogenes such as *c-kit*, which increases cellular proliferation in several malignant tumors [9,10]. 

Hence, cancer proliferation can be hampered by small molecules that are capable of easing G-quadruplex formation and/or stabilizing its structure.

Among all of the existing natural classes of substances, alkaloids have recently drawn huge attention due to their potential therapeutic employments because they present high efficacy and low systemic toxicity. As a matter of fact, these compounds possess anticancer, antiasthma, antiviral, antiarrhythmic, analgesic, antibacterial, antihyperglycemic, and antimalarial activities [11,12,13,14,15]. 

**Figure 1:**
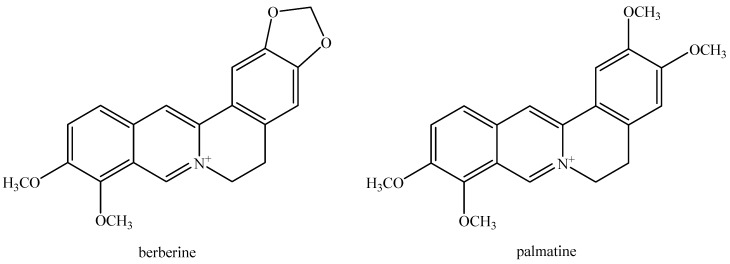
Structures of berberine and palmatine.

Within this class, berberine and palmatine (Figure 1) (derived respectively from *Berberis vulgaris* L. [16] and *Coptis japonica* (Thunb.) Makino [17]), represent the two major compounds that have been shown to strongly inhibit telomere elongation and stabilize GqS [18,19]. From the chemical standpoint, they are both quaternary ammonium salts, but berberine is a benzylisoquinoline alkaloid, whilst palmatine is an isoquinoline alkaloid. 

Previous interaction studies of these compounds have clearly revealed their weak interaction with DNA as well as their low selectivity and thermodynamic stability with GqS [20,21]. For these reasons, several efforts have been undertaken to improve their ability to stabilize G-quadruplex structures [22,23,24,25]. 

Our research group has also focused its attention on this matter, adopting a chemical–structural approach. In fact, it has been widely proven that the combination of specific peptides with specific DNA binding scaffolds greatly increases their selectivity towards GqS versus duplex DNA. In particular, this selectivity is much higher if short oligonucleotides are used, because they can favor the linkage to the complementary DNA bases that become accessible after the formation of G-quadruplex structures [26].

First, we developed one berberine derivative that was characterized by the presence of one further side chain linked to the base structure [23,27]. Its synthetic pathway was extremely simple. Moreover, PAGE analysis, using the telomeric single-stranded DNA TSG4, showed that this compound was able to increase the stability of GqS more than berberine alone [23]. However, the same compound was able to induce only a modest increase of the melting temperature (ΔT_m_ << 1 °C), as shown by a fluorescence resonance energy transfer (FRET) analysis [23]. 

For this reason, we began to study palmatine and its derivatives, believing that isoquinoline alkaloids might show a higher affinity and specificity for GqS. In this work, the design, synthesis, and spectroscopic characterization of several palmatine derivatives developed in our laboratory are reported for the first time, as well as the preliminary evaluations of their efficacy as stabilizers of GqS. Several side chains have been taken into account, and an initial comparison among the different results has also been performed. The final aim of this work was to provide a general overview and analysis of the possible synthesis and interaction of compounds coming from natural sources that might show potential antitumoral activities by acting on GqS.

## 2. Results and Discussion

**Scheme 1:**
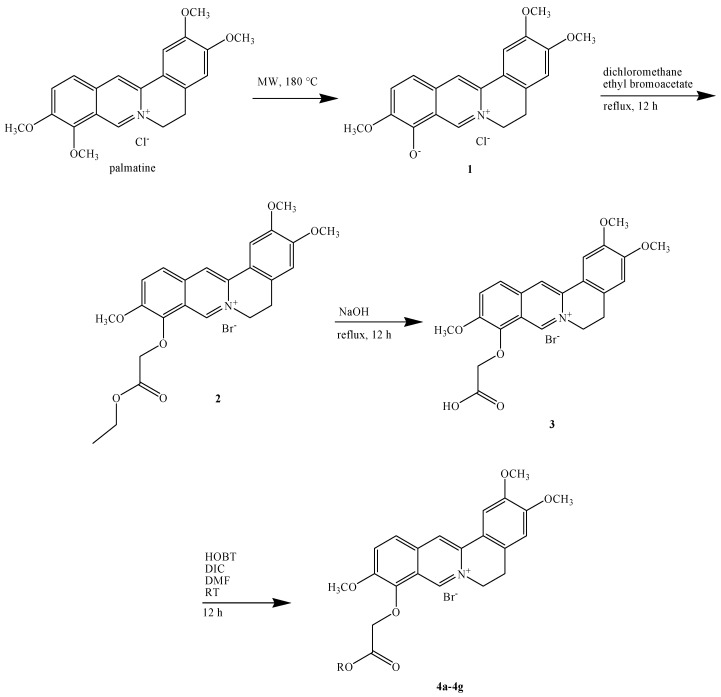
Synthesis of the 9-peptidyl palmatine derivatives (**4a**–**4g**).

Scheme 1 displays the synthetic pathway followed for the preparation of the 9-peptidyl-palmatine derivatives.

With respect to the synthetic scheme followed for berberine derivatives as reported in the work by Franceschin et al. [23], different side chains were utilized. In particular, these peptide side chains are described in Table 1.

This difference between the two protocols was derived from an extensive preliminary observation of literature data. In fact, a previous study on this subject by Zhou et al. [28] widely demonstrated that (1) palmatine derivatives with side chains that had terminal amino groups were able to better stabilize GqS, thus significantly improving their inhibitory activity on telomerase, and (2) the specific presence of amino acid chains on quinolidine structures deeply increased the ability of these compounds to interact with GqS. These two factors proved to be extremely important, because the work has given the model to follow in order to achieve the desired aim. The second conclusion, in particular, represented the fundamental basis for our work. Indeed, the ring system of palmatine closely resembles that of quindoline, and it might be expected that similar situations may lead to similar results. So, several palmatine derivatives with peptide side chains were prepared also in order to evaluate their single eventual contributions on the total effects. 

The ability of the palmatine derivatives (compounds **4a**–**4g**) to bind and stabilize GqS was evaluated by several studies. First of all, in order to study the influence of the peptide side chains on the palmatine scaffold for the different compounds, molecular docking assays (MDA) were performed on four different parallel G-qradruplex structures formed by DNA sequences that have been thoroughly studied in the literature: three are comprised in oncogene promoters, Bcl2, c-Kit1, and c-Myc [29,30]; whereas hTG21 [23] contains human telomeric G-rich repeats. The results were reported in Table 2, and showed that, in general, compounds (**4a**–**4g**) possess a higher affinity of linkage with G-quadruplex than palmatine alone. 

However, the same compounds also showed a flat SAR on the different DNA strands, but this effect could be fully explained by the well-known limitation of the technique itself. Anyway, these values are indicative on a relative scale, and are useful for studying the behavior of similar compounds [31]. Moreover, our results showed good accordance with the model that was already present in the literature [32].

**Figure 2:**
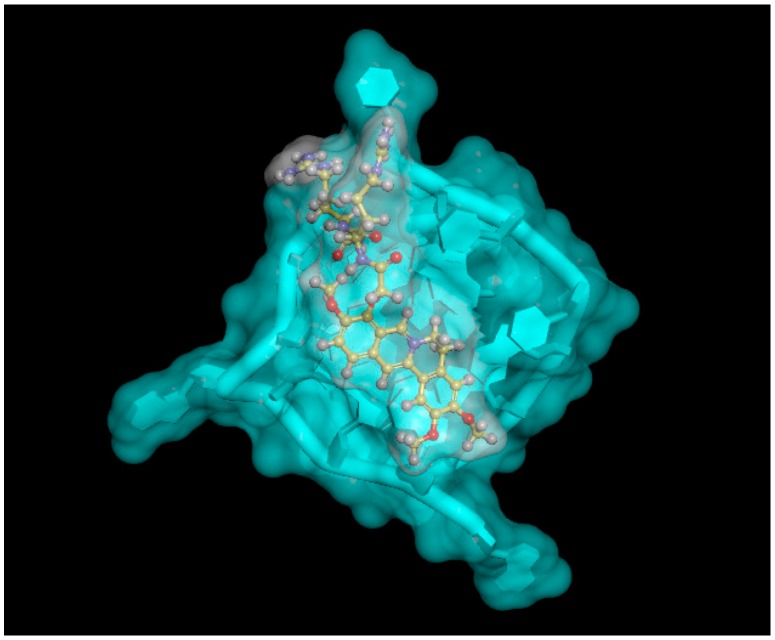
Interaction between DNA G-Quadruplex (C-Kit1) and **4a**. Blue = DNA; yellow = Carbon; dark blue = Nitrogen, red = Oxygen.

Figure 2 shows the specific interaction between the ligand (**4a**) and c-Kit1. In this case, it was possible to observe that the core of the ligand interacts only with the aromatic area of the G-tetrad, while the side chain (RR) is inserted in the groove of the DNA, thus confirming the hypothesis by Zhou et al. [28].

All of the synthesized compounds were also evaluated for their thermodynamic stability and selectivity towards GqS with respect to duplex DNA. The fluorescence resonance energy transfer (FRET) melting technique was used. For this assay, the used G-quadruplex sequences were Fc-*kit*1T, FPu18T, F21T and F10T. The sequences of these oligonucleotides are reported in Table 3. 

These oligonucleotides were chosen because Fc-*kit*1T represents the c-*kit* promoter DNA sequence, FPu18T represents the oncogene c-myc promoter DNA sequence, F21T contains human G-rich telomeric repeats, and F10T is a self-complementary duplex DNA hairpin representing a typical non-quadruplex control [33]. 

Table 4 below shows the results for the palmatine derivatives (**4a**–**4g**).

The analysis revealed that all of these ligands were really able to increase the stabilization of the G-quadruplex sequence with a wide range of ΔT_m_ values. In particular, the stabilizing activity of the tested compounds was higher towards Fc-*kit*-1T and FPu18T G-quadruplexes than toward the telomeric G-quadruplex F21T. In fact, the ΔT_m_ values of the tested compounds relative to these oligonucleotides showed interesting and extraordinary ranges from 6.1 °C to 21.7 °C for Fc-*kit*-1T, and 5.0 °C to 23.8 °C for FPu18T, whereas the telomeric G-quadruplex itself had a ΔT_m_ value ranges from 0.0 °C to 11.0 °C. As a consequence, the efficacy of the stabilizing activity of the ligands on these sequences were clearly proven. 

By comparison of the FRET results among the different synthesized ligands, it was possible to observe that compounds **4a**, **4b**, **4c** with dipeptidyl side chains were better stabilizers than compounds **4d**, **4e**, **4f**, **4g**, which presented a further amino acid residue on the peptide chains. This observation allowed us to establish, from an experimental point of view, that the side chains with more than two amino acids on palmatine derivatives did not increase the ability of the compound to stabilize GqS, but rather had the opposite effect. In fact, they greatly reduced it. 

It is very likely that this depends on the size of the loop and the different ability of the ligand to be accommodated with its peptide basic side chain in the GqS, which is absolutely essential for the stabilization of the entire structure. However, an important gain in the selectivity towards the G-quadruplex with respect to the duplex for most of the synthesized compounds was observed. 

After that, the microscale thermophoresis assay (MST) was used to further investigate the binding affinity of ligands for Fc-*kit*1T. In addition, the same technique was used to investigate their level of specificity for Fc-*kit*1T against duplex DNA. In fact, MST is a highly sensitive technique that acts as a real probe for many kinds of binding-induced interactions such as molecular size, charge, hydration shell, or conformation [34], and possesses several advantages, including easy implementation, high speed (about 1 min), low sample requirements (e.g., microliter volumes), no limitations on the molecular size or weight of the binding partners, and the possibility of using several buffer solutions [35].

Table 5 and Table 6 show the results of this analysis.

As shown, all of the tested ligands were found to bind to Fc-*kit*1T more tightly than duplex DNA. The dissociation constants (K_d_) for Fc-*kit*1T were between 1.41 μM and 11.1 μM against 10.7–45.5 μM for the duplex DNA. From these data, it could be clearly deduced that the introduction of peptidyl side chains on the scaffold of palmatine gave a moderate improvement of its specificity towards the quadruplex with respect to duplex DNA with a good increase of the *K*_d_^duplex^/*K*_d_^Fc-*kit*1T^starting from the value of 1.6 associated to palmatine. In particular, the ligands **4a**, **4b**, **4c** with *K*_d_^duplex^/*K*_d_^Fc-*kit*1T^ values ranging from 7.6 to 13.9 showed higher affinity and selectivity towards Fc-*kit*1T than the ligands **4d**, **4e**, **4f**, **4g**, which had *K*_d_^duplex^/*K*_d_^Fc-*kit*1T^ values ranging from 2.1 to 4.2. Yet, it was not possible to verify whether this result was part of a general tendency, because only one sequence was completely studied. Further studies in this field should be performed.Anyway, these preliminary results were in good accordance with those obtained from FRET analysis, thus making all of these ligands potential good compounds for the stabilization of GqS and, by consequence, potential good antitumor agents. 

## 3. Materials and Methods

### 3.1. Materials

Pure palmatine was purchased from Sigma-Aldrich () (Darmstadt, Germany) as well as the other natural solvents of RPE purity grade, if not diversely specified, and the deuterated solvents. The Fmoc-protected amino acids and Rink amide AM resin were purchased from GL Biochem (Shanghai, China) Ltd. All of the oligomers/primers were purchased from Invitrogen and Sangon Biotech (Shanghai, China). Stock solutions of all of the derivatives (10 mM) were made using DMSO (10%) or double-distilled deionized water. Silica gel for classical column chromatography (40–63 μm particle size) was purchased from Fluka Analytical (St. Gallen, Switzerland), while silica gel for flash column chromatography (200–300 mesh, was purchased from Qingdao Haiyang Chemical Co. Ltd. (Qingdao, Shandong, China).

### 3.2. Instrumentation 

^1^H-NMR and ^13^C-NMR spectra were recorded on a Varian (now Agilent Technologies, Santa Clara, CA, USA) Mercury 300 MHz spectrometer and/or on a Bruker BioSpin GmbH 400 MHz spectrometer (Billerica, MA, USA) using CD_3_OD and DMSO-*d*_6_ as deuterated solvents. The chemical shifts were expressed from the internal solvent signal of CD_2_HOD (m5, 3.31 ppm) for spectra in CD_3_OD, while the internal solvent signal of D_3_SOD_3_ (p, 2.50 ppm) was the reference for spectra in DMSO-*d*_6_.

Mass spectra were, instead, recorded on a Shimadzu LCMS-2010A instrument or on a Q-TOF MICRO spectrometer (Micromass, now Waters, Manchester, UK) instrument. The first instrument used an ESI-API (electrospray ionization–atmospheric pressure ionization) source, whereas the second instrument used only an ESI source. Both instruments operated in positive ionization with a voltage of 5000 V. The flow rates of sample infusion for both instruments were 30 μL/min, with 50 acquisitions per spectrum. Data coming from both instruments were analyzed by using the MassLynx software developed by Waters. High-resolution mass spectra (HRMS) were performed on a Shimadzu LCMS-IT-TOF instrument (Kyoto, Japan) with the same characteristics as previously described.

The purification of the reaction mixtures was achieved by means of classical column chromatography on silica gel, by means of flash column chromatography or by means of inverse column chromatography with stationary phase (C18)-bonded silica (USP classification L1). The purification of all of the tested compounds was carried out through a preparative HPLC using a Varian Pro Star equipped with an UV-vis detector (BioRad, Santa Clara, CA, USA) and a Phenomenex C18 (10.0 × 250 mm, 5 μm) column.

The microwave reaction was performed with Biotage Initiatior (Biotage, Uppsala, Sweden). 

The radioactivity was quantified by using a Beckman LS 5000 TD Scintillation System (Dickinson, TX, USA).

### 3.3. Synthesis of the Peptide Side Chains

Peptide side chains were synthesized by using a solid-phasemethodology through a manual operation of the peptide synthesizer. The Fmoc protected various amino acids (four equivalent ratio excess to the resin) were coupled orderly to the Rink Amide AM resin (500 mg) by using DIC/HOBT (190 mg/200 mg) in DMF (4 mL) for 3 h. The deprotection of the Fmoc group was carried out with piperidine/DMF 25% (2 mL) for 30 min. 

### 3.4. Synthesis of Palmatine Derivatives 

The synthesis of the palmatine derivatives (compounds **4a**–**4g**) was achieved by using a similar procedure as reported in the literature for berberine [22], but with some modifications. In particular, the first step was optimized for the microwave reaction by increasing the quantity of the reagent, while the purification was performed on an inverse chromatographic column by using a mixture of CH_3_OH/H_2_Owith 1% of TFA from 5:95 (*v*/*v*) to 1:1 (*v*/*v*).

***Reaction 1:***The reaction was performed under microwave irradiation. A quantity of palmatine for the weight of 2.0 g (5.15 mmol) was added in a flask provided with a stir bar. The solution was pre-stirred for 1 min before raising the temperature. The machine was programmed to high levels of absorption. The solution was heated to 180 °C, with a voltage of 400 W, for 5–10 min. The reaction gave a red precipitate, compound (**1**), which was washed and filtered with ether under vacuum with a final yield of 95%.

***Reaction 2:***Compound (**1**) present in CH_2_Cl_2_ was treated with the ethyl ester of bromoacetic acid in a ratio of 1:2 (*v*/*v*), stirred on a magnetic stirrer, and refluxed for 12 h, as suggested in the literature [36]. The resulting yellow precipitate was filtered off, refluxed with EtOH for 30 min, cooled, filtered off again, and dried in a vacuum to give compound (**2**) with a yield of 55%.

***Reaction 3:***Compound (**2**) was dissolved in a solution of CH_3_OH/H_2_O (1:1 (*v*/*v*)). Then, NaOH in a ratio of 1:2 (*w*/*w*) was added under reflux for 12 h on a water bath, and the solution was cooled [36]. At this point, HCl was added until a pH value of 1 was obtained. The resulting yellow precipitate was filtered off and dried in a vacuum to give compound (**3**) with a yield of 45%.

***Reaction 4:***To have compounds (**4a**–**4g**), the intermediate compound (**3**) for the weight of 200 mg was mixed with deprotected peptidyl side chains using DIC/HOBT (475 mg/500 mg) in DMF (4 mL) with shaking for 12 h. After coupling with compound (**3**), the resin was treated with the cleavage reagent (95% TFA, 5% dd H_2_O (*v*/*v*)) for 2 h, washed with dry ether (30 mL), and then evaporated under vacuum. The crude products were purified by using preparative RP-HPLC with CH_3_CN/H_2_O (95:5 to 5:95) containing 0.1% TFA at a flow rate of 2.5 mL/min elution, and then lyophilized to give the final compounds. For compound (**4a**), the peptidyl coupling order is lysine-arginine, and this was a yellow solid with a yield of 10%. For compound (**4b**), the peptidyl coupling order was phenylalanine-arginine, and it was a yellow solid with a 29% yield. For compound (**4c**), the peptidyl coupling order was arginine–arginine, and it was a yellow solid with a 5% yield. For compound (**4d**), the peptidyl coupling order was phenylalanine–histidine–arginine, and it was a yellow solid with a 5% yield. For compound (**4e**), the peptidyl coupling order was lysine-phenylalanine-arginine–, and it was a yellow solid with a 10% yield. For compound (**4f**), the peptidyl coupling order was phenylalanine-glycine-lysine, and it was a yellow solid with an 8% yield. For compound (**4g**), the peptidyl coupling order was phenylalanine-lysine-arginine e, and it was a yellow solid with a 10% yield. 

### 3.5. NMR and MS Data of Compounds (* = IUPAC Names)

**Figure 3:**
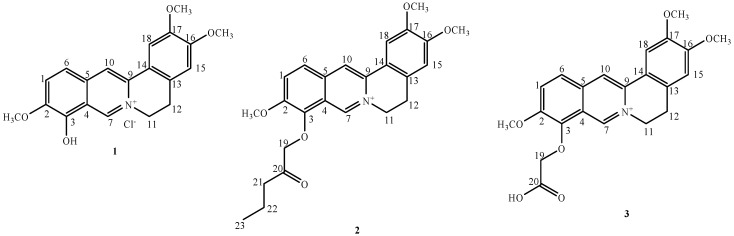
Structures of compounds (**1**–**3**).

*3-Hydroxy-2,16,17-trimethoxy-11,12-dihydroisoquinolino[9,8-a]isoquinolin-7-ium chloride* (**1**) (Figure 3): ^1^H-NMR (400 MHz, DMSO) δ: 9.11 (1H, s, H-7), 8.08 (1H, s, H-10), 7.52 (1H, s, H-18), 7.24 (1H, t, *J* = 6.4 Hz, H-6), 6.99 (1H, s, H-15), 6.40 (1H, d, *J* = 6.4 Hz, H-1), 4.50 (2H, t, *J* = 4.4 Hz, H-11), 3.90 (3H, s, OCH_3_-17), 3.83 (3H, s, OCH_3_-16), 3.74 (3H, s, OCH_3_-2), 3.07 (2H, t, *J* = 4.4Hz, H-12). ESI-API-MS: *m*/*z* 338.1 [M]^+^ (calculated for C_20_H_20_NO_4_, 338.4 [M]^+^).

*3-(2-Ethoxy-2-oxoethoxy)-2,16,17-trimethoxy-11,12-dihydroisoquinolino[9,8-a]isoquinolin-7-ium* (**2**) (Figure 3): ^1^H-NMR (400 MHz, DMSO) δ: 9.93 (1H, s, H-7), 9.02 (1H, s, H-10), 8.20 (1H, d, *J* = 8.2 Hz, H-6), 8.02 (1H, d, *J* = 8.2 Hz, H-1), 7.71 (1H, s, H-18), 7.10 (1H, s, H-15), 5.06 (2H, s, H-19), 4.94 (2H, t, *J* = 4.9 Hz, H-11), 4.17 (2H, q,*J* = 4.1 Hz, H-21), 4.03 (3H, s, OCH_3_-17), 3.93 (3H, s, OCH_3_-16), 3.87 (3H, s, OCH_3_-2), 3.22 (2H, t, *J* = 4.9 Hz, H-12), 1.19 (3H, t, *J* = 4.1 Hz, H-23). ESI-API-MS: *m*/*z* 424.3 [M]^+^ (calculated for C_24_H_27_NO_6_, 424.5 [M]^+^).

*3-(Carboxymethoxy)-2,16,17-trimethoxy-11,12-dihydroisoquinolino[9,8-a]isoquinolin-7-ium* (**3**) (Figure 3): ^1^H-NMR: (400 MHz, DMSO) δ: 10.40 (1H, s, H-7), 8.98 (1H, s, H-10), 7.59 (1H, d, *J* = 8.2 Hz, H-6), 7.49 (1H, d, *J* = 8.2 Hz, H-1), 7.43 (1H, s, H-18), 6.94 (1H, s, H-15), 4.87 (2H, t, *J* = 4.9 Hz, H-11), 4.26 (2H, s, H-19), 3.88 (3H, s, OCH_3_-17), 3.87 (3H, s, OCH_3_-16), 3.82 (3H, s, OCH_3_-2), 3.22 (2H, t, *J* = 4.9 Hz, H-12).ESI-API-MS: *m*/*z* 396.1 [M]^+^ (calculated for C_22_H_23_NO_6_, 396.4 [M]^+^).

**Figure 4:**
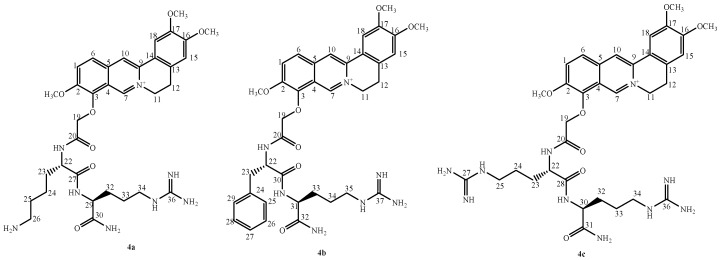
Structures of compounds (**4a**–**4c**).

3-(2-(((S)-6-Amino-1-(((S)-1-amino-5-guanidino-1-oxopentan-2-yl)amino)-1-oxohexan-2-yl)amino)-2-oxoethoxy)-2,3,10-trimethoxy-5,6-dihydroisoquinolino[3,2-a]isoquinolin-7-ium* (**4a**) (Figure 4): ^1^H-NMR (400 MHz, CD_3_OD) δ: 9.95 (1H, s, H-7), 8.83 (1H, s, H-10), 8.16 (1H, d, J = 8.0 Hz, H-6), 8.14 (1H, d, J = 8.0 Hz, H-1), 7.68 (1H, s, H-18), 7.06 (1H, s, H-15), 6.09 (2H, t, J = 6.1 Hz, H-11), 4.39 (1H, m, H-22), 4.26 (1H, m, H-29), 4.13 (3H, s, OCH_3_-17), 4.07 (2H, m, H-34), 4.00 (3H, s, OCH_3_-16), 3.95 (3H, s, OCH_3_-2), 3.22 (2H, t, J = 6.0 Hz, H-12), 3.20 (2H, m, H-26), 1.71 (10H, m, H-23, H-24, H-25, H-32, H-33). HR-MS(LC-MS-IT-TOF): *m*/*z* 580.6512 [M]^+^.

9-(3-(((S)-1-(((S)-1-Amino-6-guanidino-1-oxohexan-2-yl)amino)-1-oxo-3-phenylpropan-2-yl)amino)-3-oxopropyl)-2,3,10-trimethoxy-5,6-dihydroisoquinolino[3,2-a]isoquinolin-7-ium* (**4b**) (Figure 4): ^1^H-NMR (400 MHz, CD_3_OD) δ: 9.96 (1H, s, H-7), 8.72 (1H, s, H-10), 8.14 (1H, d, J = 8.0 Hz, H-6), 8.05 (1H, d, J = 8.0 Hz, H-1), 7.66 (1H, s, H-18), 7.15 (5H, m, H-25, H-26, H-27, H-28, H-29), 7.05 (1H, s, H-15), 6.11 (2H, t, J = 6.0 Hz, H-11), 4.95 (2H, s, H-19), 4.47 (1H, m, H-31), 4.13 (3H, s, OCH_3_-17), 4.12 (3H, s, OCH_3_-16), 4.09 (3H, s, OCH_3_-2), 4.07 (2H, m, H-35), 3.16 (2H, d, J = 6.0 Hz, H-12), 1.71 (4H, m, H-23, H-33). HR-MS(LC-MS-IT-TOF): *m*/*z* 698.3296 [M]^+^.

3-(2-(((S)-5-((1-Aminovinyl)amino)-1-oxo-1-(((S)-2-oxo-6-(prop-1-en-2-ylamino)hexan-3-yl)amino)pentan-2-yl)amino)-2-oxoethoxy)-2,3,10-trimethoxy-5,6-dihydroisoquinolino [3,2-a]isoquinolin-7-ium* (**4c**) (Figure 4): ^1^H-NMR (400 MHz, CD_3_OD) δ: 9.96 (1H, s, H-7), 8.72 (1H, s, H-10), 8.14 (1H, d, J = 8.0 Hz, H-6), 8.05 (1H, d, J = 8.0 Hz, H-1), 7.66 (1H, s, H-18), 7.05 (1H, s, H-15), 6.04 (2H, t, J = 6.0 Hz, H-11), 4.95 (2H, s, H-19), 4.69 (1H, m, H-30), 4.47 (1H, m, H-22), 4.13 (3H, s, OCH_3_-17), 4.12 (3H, s, OCH_3_-16), 4.09 (3H, s, OCH_3_-2), 4.07 (4H, m, H-25, H-34), 3.16 (2H, d, J = 6.0 Hz, H-12), 1.87 (4H, m, H-23, H-32), 1.71 (4H, m, H-24, H-33). HR-MS(LC-MS-IT-TOF): *m*/*z* 707.3623 [M]^+^. 

**Figure 5:**
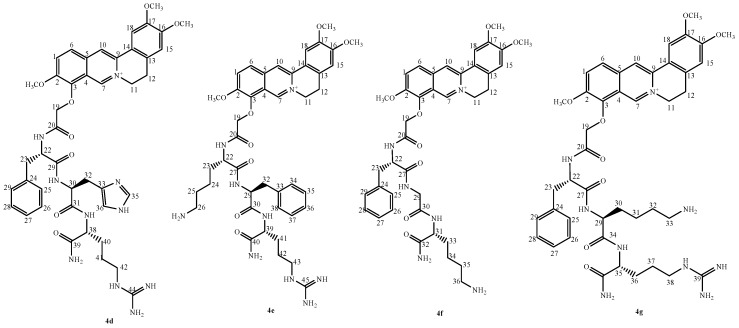
Structures of compounds (**4d**–**4g**).

9-(2-(((S)-1-(((S)-1-(((S)-1,6-Diamino-1-oxohexan-2-yl)amino)-3-(1H-imidazol-4-yl)-1-oxopropan-2-yl)amino)-1-oxo-3-phenylpropan-2-yl)amino)-2-oxoethoxy)-2,3,10-trimethoxy-5,6-dihydroisoquinolino[3,2-a]isoquinolin-7-ium* (**4d**) (Figure 5): ^1^H-NMR: (400 MHz, CD_3_OD) δ: 9.89 (1H, s, H-7), 8.78 (1H, s, H-10), 8.16 (1H, d, J = 8.0 Hz, H-6), 8.10 (1H, d, J = 8.0 Hz, H-1), 7.99 (1H, s, H-36), 7.70 (1H, s, H-18), 7.20 (5H, m, H-25, H-26, H-27, H-28, H-29), 7.08 (1H, s, H-15), 7.05 (1H, s, H-35),6.09 (2H, t, J = 6.0 Hz, H-11), 4.95 (2H, s, H-19), 4.71 (1H, m, H-30), 4.54 (1H, m, H-22), 4.36 (1H, m, H-38), 3.97 (3H, s, OCH_3_-17), 3.93 (3H, s, OCH_3_-16), 3.88 (3H, s, OCH_3_-2), 3.91 (2H, s, H-32), 3.79 (2H, m, H-23), 3.20 (2H, m, H-42), 3.18 (2H, t, J = 6.0 Hz, H-12), 1.90 (2H, m, H-41), 1.71 (2H, m, H-40). HR-MS(LC-MS-IT-TOF): *m*/*z* 835.9367 [M]^+^.

9-(((6S,9S,12S)-1-Amino-12-(4-aminobutyl)-9-benzyl-6-carbamoyl-1-imino-8,11,14-trioxo-2,7,10,13-tetraazapentadecan-15-yl)oxy)-2,3,10-trimethoxy-5,6-dihydroisoquinolino[3,2-a]isoquinolin-7-ium* (**4e**) (Figure 5): ^1^H-NMR (400 MHz, CD_3_OD) δ: 9.96 (1H, s, H-7), 8.73 (1H, s, H-10), 8.13 (1H, d, J = 8.0 Hz, H-6), 8.05 (1H, d, J = 8.0 Hz, H-1), 7.50 (1H, s, H-18), 7.22 (5H, m, H-34, H-35, H-36, H-37, H-38), 7.12 (1H, s, H-15), 6.05 (2H, t, J = 6.0 Hz, H-11), 4.96 (2H, s, H-19), 4.81 (1H, m, H-29), 4.74 (1H, m, H-22), 4.66 (1H, m, H-39), 4.02 (3H, s, OCH_3_-17), 4.01 (3H, s, OCH_3_-16), 3.98 (3H, s, OCH_3_-2), 3.81 (2H, m, H-43), 3.27 (2H, m, H-32), 3.21 (2H, m, H-23), 3.20 (2H, m, H-26), 3.03 (2H, t, J = 6.0 Hz, H-12), 1.90 (4H, m, H-42), 1.71 (2H, m, H-24, H-41), 1.65 (2H, m, H-25). HR-MS (LC-MS-IT-TOF): *m*/*z* 826.4581 [M]^+^.

9-(2-(((S)-1-((2-(((S)-1,6-Diamino-1-oxohexan-2-yl)amino)-2-oxoethyl)amino)-1-oxo-3-phenylpropan-2-yl)amino)-2-oxoethoxy)-2,3,10-trimethoxy-5,6-dihydroisoquinolino[3,2-a]isoquinolin-7-ium* (**4f**) (Figure 5): ^1^H-NMR (400 MHz, CD_3_OD) δ: 9.94 (1H, s, H-7), 8.74 (1H, s, H-10), 8.13 (1H, d, J = 8.0 Hz, H-6), 8.06 (1H, d, J = 8.0 Hz, H-1), 7.75 (1H, s, H-18), 7.23 (5H, m, H-25, H-26, H-27, H-28, H-29), 7.12 (1H, s, H-15), 6.04 (2H, t, J = 6.0 Hz, H-11), 4.92 (2H, s, H-19), 4.70 (1H, m, H-31), 4.41 (1H, m, H-22), 4.09 (2H, s, H-29), 3.99 (3H, s, OCH_3_-17), 3.97 (3H, s, OCH_3_-16), 3.94 (3H, s, OCH_3_-2), 3.61 (2H, m, H-36), 3.13 (2H, t, J = 6.0 Hz, H-12), 3.08 (2H, m, H-23), 1.92 (2H, m, H-34), 1.70 (2H, m, H-33), 1.45 (2H, m , H-35). HR-MS(LC-MS-IT-TOF): *m*/*z* 727.8355 [M]^+^.

9-(((6S,9S,12S)-1-Amino-9-(4-aminobutyl)-12-benzyl-6-carbamoyl-1-imino-8,11,14-trioxo-2,7,10,13-tetraazapentadecan-15-yl)oxy)-2,3,10-trimethoxy-5,6-dihydroisoquinolino[3,2-a]isoquinolin-7-ium* (**4g**) (Figure 5): ^1^H-NMR (400 MHz, CD_3_OD) δ: 9.98 (1H, s, H-7), 8.69 (1H, s, H-10), 8.15 (1H, d, J = 8.0 Hz, H-6), 8.04 (1H, d, J = 8.0 Hz, H-1), 7.51 (1H, s, H-18), 7.27 (5H, m, H-25, H-26, H-27, H-28, H-29), 7.12 (1H, s, H-15), 6.04 (2H, t, J = 6.0 Hz, H-11), 4.94 (2H, s, H-19), 4.80 (1H, m, H-22), 4.73 (1H, m, H-29), 4.69 (1H, m, H-35), 4.05 (3H, s, OCH_3_-17), 4.06 (3H, s, OCH_3_-16), 3.95 (3H, s, OCH_3_-2), 3.71 (2H, m, H-38), 3.21 (2H, m, H-23), 3.20 (2H, m, H-33), 3.02 (2H, t, J = 6.0 Hz, H-12), 1.90 (4H, m, H-32, H-37), 1.70 (4H, m, H-31, H-36), 1.65 (2H, m, H-30). HR-MS(LC-MS-IT-TOF): *m*/*z* 826.4537 [M]^+^. 

### 3.6. Structural and Biological Analysis 

#### 3.6.1. Molecular Docking

The crystal structures of G-quadruplex DNA were extracted from the Protein Data Bank (PDB; PDB) and are reported in Table 7. 

The structures were treated with the Maestro module of Schrödinger Suite 2012 as follows: all of the sulfate ions and water molecules found in the structure were removed, hydrogen atoms were added, and the H-bond assignment was optimized with exhaustive sampling.

Ligand structures were constructed and optimized by using the Maestro 9.3 followed by rebuilding the three-dimensional (3D) conformation of each molecule using LigPrep 2.3 at pH of 7.0. Afterwards, the rebuilt ligands were subjected to a conformational search using Macromodel 9.7 with an OPLS 2005 force field in a water solvent model. For each DNA, first, a grid box of 36 × 36 × 36 Å with a default inner box (10 × 10 × 10 Å) was centered on the DNA itself. 

For all of the experiments, the standard precision mode of GlideScore was selected as the scoring function. The top 10 poses were kept, and XP score functions between the ligands and the DNA were analyzed.

#### 3.6.2. FRET Assay 

FRET assay was carried out on a real-time PCR apparatus following previously published procedures [37]. 

The fluorescently-labeled oligonucleotides used as FRET probes (F21T, Fc-kit1T, FPu18T, the donor fluorophore 6-carboxyfluorescein (FAM), and the acceptor fluorophore 6-carboxytetramethylrhodamine (TAMRA)) were diluted from stock to the correct concentration (800 nM) in a 0.5-mM potassium cacodylate buffer (pH 7.4) with 100 mM of LiCl, and then annealed by heating to 85 °C for 10 min, followed by slowly cooling down to room temperature in the heating block.

Fluorescence melting curves were determined with a Roche LightCycler 2 real-time PCR apparatus, using a total reaction volume of 20 μL with 0.2 μM of fluorescently labeled oligonucleotides (prepared as described before) in the presence or absence of 2 μM of every compound. Fluorescence readings with excitation at 470 nm and detection at 530 nm were taken at intervals of 1 °C over the range 37–99 °C, with a constant temperature being maintained for 30 s prior to each reading to ensure a stable value. Final analysis of the data was carried out using Origin8.0 (OriginLab Corp, Northampton, MA, USA).

#### 3.6.3. MST Assay 

The fluorescently labeled oligonucleotides were diluted from stock to the required concentration (10 µM) in 10 mM of Tris-HCl buffer, pH 7.4, in the presence of 100 mM of KCl, and then annealed by heating at 95 °C for five minutes, gradually cooled to room temperature, and incubated at 4 °C overnight. The ligand concentration varied from 0 μM to 200 μM for tested the *K*_D_ of duplex DNA. A 16-point dilution series was prepared for each DNA. After incubation, the samples were loaded into MST standard-treated glass capillaries. The binding affinity study was investigated on the microscale thermophoresis instrument (NanoTemper Tchnologies GmbH, Munich, Germany). The value of *K*_d_ was calculated by NT ANALYSIS SOFTWARE provided by NanoTemper Technologies GmbH (Munich, Germany).

## 4. Conclusions

The chemical synthesis of seven new palmatine derivatives was successfully carried out with few steps and not bad yields. The preliminary FRET and MST assays clearly showed how the further presence of side chains on the core structure of these compounds is important in order to improve their interaction with GqS and increase the stability of the complex structures formed. This latter aspect is extremely important, because this may represent the base of action to treat several tumors. Therefore, the future perspectives are promising for the hope to finally find the perfect compound with the best anticancer activity.

## Figures and Tables

**Table 1:** Structures of the synthesized 9-peptidyl palmatine derivatives (**4a**–**4g**).

Ligand	Peptide Side Chain
**4a**	K-R
**4b**	F-R
**4c**	R-R
**4d**	F-H-R
**4e**	K-F-R
**4f**	F-G-K
**4g**	F-K-R

G = Glycine; R = Arginine; K = Lysine; H = Histidine; F = Phenylalanine.

**Table 2:** Docking results for palmatine and palamtine derivatives.

LIGAND	Bcl-2	c-Kit1	c-Myc	hTG21
**4a**	−8.4	−10.7	−11.1	−10.7
**4b**	−8.2	−10.3	−11.1	−9.3
**4c**	−9.0	−10.9	−10.8	−11.6
**4d**	−9.8	−10.8	−11.7	−11.5
**4e**	−9.7	−11.3	−10.9	−11.3
**4f**	−7.4	−10.3	−10.5	−10.5
**4g**	−8.9	−10.9	−11.8	−12.0
Palmatine	−2.5	−3.6	−3.09	−2.9

Compounds were docked on four different DNA strands: Bcl-2, C-Kit1, Pu22 (c-Myc), hTG21, all in parallel conformation. Results are express in Kcal/mol.

**Table 3:** Specifications of the sequence of the oligonucleotides used for the fluorescence resonance energy transfer (FRET) analysis.

Oligonucleotide	Sequence
Fc-*kit*1T	5′-FAM-d[AGGGAGGGCGCTGGGAGGAGGG]-TAMRA-3′
FPu18T	5′-FAM-d[AGGGTGGGGAGGGTGGGG]-TAMRA-3′
F21T	5′-FAM-dGGGTTAGGGTTAGGGTTAGGG-TAMRA-3
F10T	5′-FAM-d[TATAGCTATA-HEG-TATAGCTATA]-TAMRA-3′

**Table 4:** G-quadruplex DNA and duplex DNA stabilization temperatures (Δ*T*_m_) obtained from FRET melting assay.

Ligands	R ^a^	FRET (ΔT_m_, °C)			
		F21T ^b^	Fc-*kit*1T ^b^	FPu18T ^b^	F10T ^b^
**4a**	KR-NH_2_	2.4 ± 0.8	12.3 ± 0.2	9.2 ± 0.5	0.4 ± 0.9
**4b**	FR-NH_2_	11.0 ± 0.1	21.7 ± 0.8	23.8 ± 0.6	0.8 ± 0.4
**4c**	RR-NH_2_	10.6 ± 0.2	21.3 ± 0.5	19.6 ± 0.9	0.3 ± 0.6
**4d**	FHR-NH_2_	0.7 ± 0.7	6.5 ± 0.2	6.0 ± 1.0	0.0 ± 1.0
**4e**	KFR-NH_2_	0.0 ± 1.2	6.5 ± 0.4	8.4 ± 0.3	0.1 ± 1.0
**4f**	FGK-NH_2_	1.0 ± 0.6	6.1 ± 0.9	5.0 ± 1.0	0.5 ± 0.1
**4g**	FKR-NH_2_	0.0 ± 1.0	6.1 ± 0.4	5.4 ± 0.2	0.1 ± 1.0
**palmatine**	--	1.8 ± 0.2	11.6 ± 0.2	11.2 ± 0.1	0.3 ± 0.3

^a^ Ris the peptidyl sequences of these conjugates side chains, starting from the terminal of scaffold. R, G, F, H, and K are abbreviations of arginine, glycine, phenylalanine, histidine, and lysine, respectively. ^b^ ΔT_m_ = *T*_m_ (DNA + ligand) − T_m_ (DNA). ΔT_m_ values of 0.2 μM F21T, Fc-*kit*1T, FPu18T, or F10T incubated with a 1.0-μM compound in the presence of 60-mM KCl.

**Table 5:** Kinetic constants of palmatine derivatives with Fc-*kit*1T and duplex DNA by microscale thermophoresis (MST) assay.

Compounds	R ^a^	Fc-*kit*1T(*K*_d_, μM)	Duplex(*K*_d_, μM)	*K*_d_^duplex^/*K*_d_*^c-kit1^*
**4a**	KR-NH_2_	2.65 ± 0.33	23.60 ± 1.07	8.9
**4b**	FR-NH_2_	1.41 ± 0.09	10.70 ± 1.30	7.6
**4c**	RR-NH_2_	1.47 ± 0.12	20.40 ± 0.96	13.9
**4d**	FHR-NH_2_	9.68 ± 0.57	40.70 ± 5.87	4.2
**4e**	KFR-NH_2_	8.00 ± 0.89	16.80 ± 1.38	2.1
**4f**	FGK-NH_2_	11.10 ± 0.51	40.70 ± 1.62	3.7
**4g**	FKR-NH_2_	6.60 ± 0.472	45.50 ± 4.57	6.9
**palmatine**	--	3.25 ± 0.32	5.08 ± 0.34	1.6

^a^ R is the peptidyl sequences of these conjugates side chains, starting from the terminal of scaffold. R, G, F, H, and K are abbreviations of arginine, glycine, phenylalanine, histidine, and lysine, respectively.

**Table 6:** Energetic values of the bond between palmatine derivatives with *c-kit1, c-Myc* and Htg21 G-quadruplex structures by MST assay.

Ligands	R ^a^	Bond Energy (kcal/mol)		
		Htg21	F*c-kit1*T	*c-Myc*
**4a**	KR-NH_2_	−10.7	−10.7	−11.8
**4b**	FR-NH_2_	−9.9	−10.3	−11.1
**4c**	RR-NH_2_	−11.3	−10.8	−10.7
**4d**	FHR-NH_2_	−11.6	−10.8	−11.3
**4e**	KFR-NH_2_	−11.3	−11.3	−10.9
**4f**	FGK-NH_2_	−10.5	−10.3	−10.5
**4g**	FKR-NH_2_	−12.0	−10.9	−11.8
**palmatine**	--	−2.9	−3.5	−3.1

^a^ Ris the peptidyl sequences of these conjugates side chains, starting from the terminal of scaffold. R, G, F, H, and K are abbreviations of arginine, glycine, phenylalanine, histidine, and lysine, respectively.

**Table 7:** G-quadruplex structures used in molecular docking. PDB: Protein Data Bank.

G-quadruplex Name	PDB ID
c-*myc* parallel	1XAV
c-*kit* parallel	2O3H
Bcl-2 parallel	2F8U
Htg21 parallel	2KZE
